# Supplementary material for: Uncovering actionable trade-offs of antifungal resistance in a yeast pathogen
Source: Mol Syst Biol. 2026 Jan 12;22(4):537–62. doi: 10.1038/s44320-025-00185-3 (PMC13046754; doi:10.1038/s44320-025-00185-3)
Supplement: Supplementary file 12 — Expanded View Figures [file 44320_2025_185_MOESM12_ESM.pdf]

Expanded View Figures

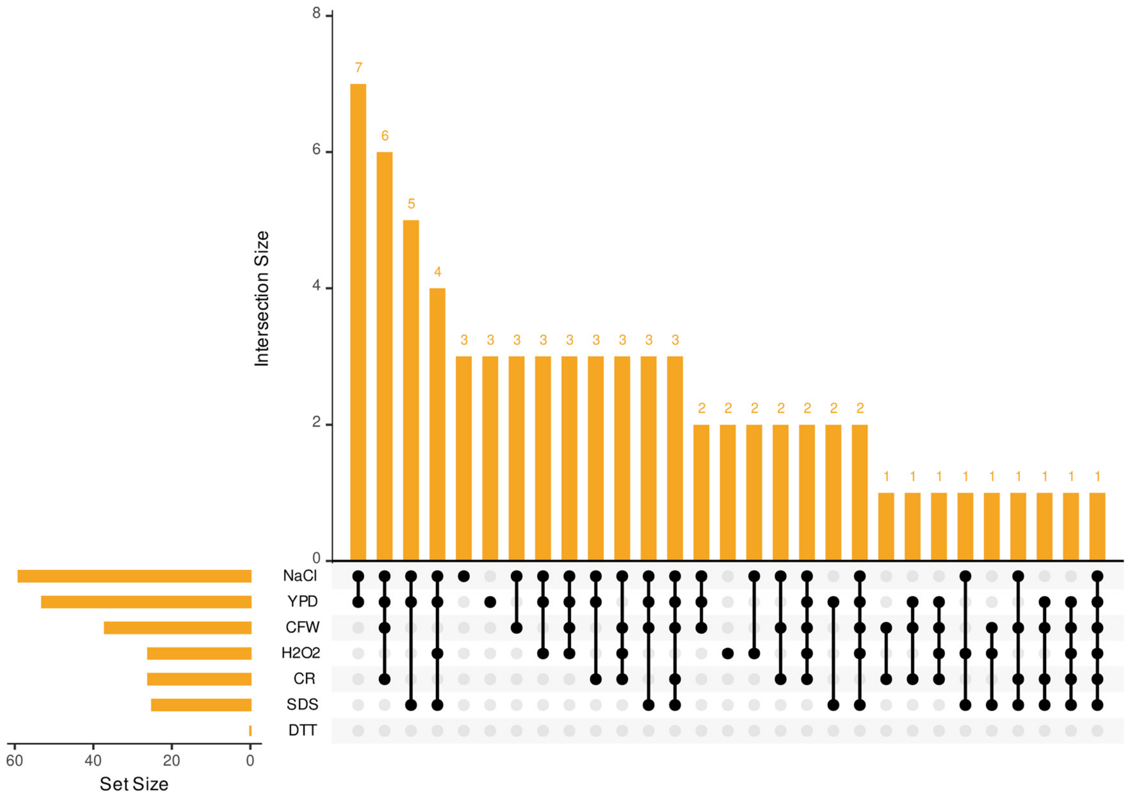

Figure EV1. Trade-off combinations observed in collection phenotyping.

Upset plot showing statistically significant negative trade-off (median fAUCR < 0.9, one-sided t-test (compared to 1) $p < 0.05$, Dataset EV4). Source data are available online for this figure.

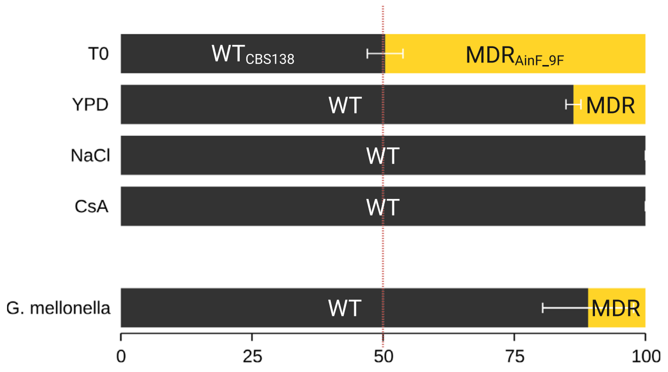

Figure EV2. Fitness trade-offs affect competitiveness of drug-resistant strains.

Results of a competition experiment of the wild type reference strain CBS138 and an multidrug resistant (MDR) strain (AinF-9F) evolved from it. Stacked bar plots showing the percentage of colonies counted after 48 h YPDa plates for each strain at the initial mix of containing 50% of each strain (T0), after 24 h growing in YPD (YPD), in YPD + 1.5 mM NaCl (NaCl), YPD + 20 µM CsA (CsA) and inside the invertebrate model of fungal infection *Galleria mellonella* (*G. mellonella*). Source data are available online for this figure.

 © The Author(s)

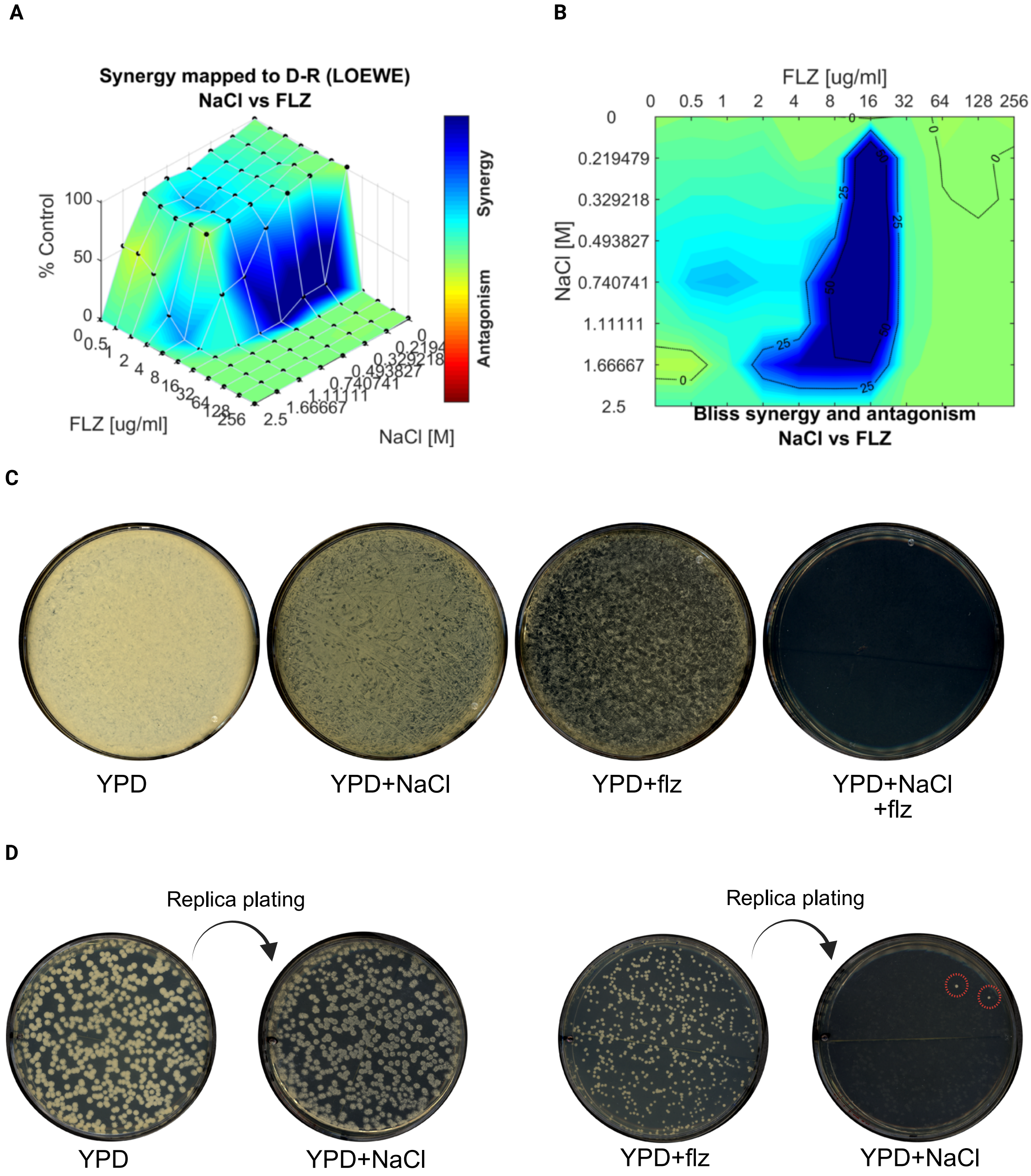

Figure EV3. Synergistic effect of NaCl with fluconazole on *N. glabratus* growth inhibition.

(**A**) 3D representation of the synergy matrix between different concentrations of fluconazole (flz) and NaCl using the Loewe synergy model. (**B**) Contour plot showing the synergy values calculated from the Bliss model. Both plots were generated by Combenefit software. (**C**) Images taken after 72 h of incubation of 10^7 cells on YPD, YPD + 1.25 M NaCl, YPD + 128 µg/mL flz, and YPD + 1.25 M NaCl + 128 µg/mL flz plates; no colonies were observed under the combined treatment. (**D**) Images of plates obtained after 72 h of replica plating onto YPD + 1.25 M NaCl (shown after the arrow) from source plates (shown before the arrow) containing ~500 colonies of YPD adapted or YPD + flz (128 µg/mL) adapted strains. On the YPD + NaCl plate replicated from YPD + flz, only two colonies were observed, marked with red circles. Source data are available online for this figure.

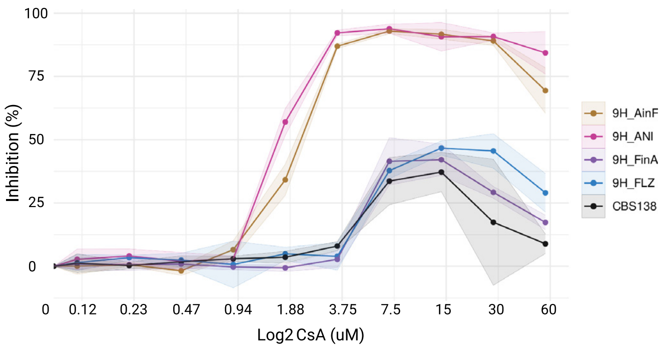

Figure EV4. Selective inhibition of Cyclosporine A to certain drug resistant strains.

Dose-response assay for Cyclosporine A (CsA) in evolved strains under different treatment of the CBS138 wild type reference strain. The y-axis indicates the percentage of growth inhibition versus control based on OD600 measurements, and the x-axis indicates CsA concentrations on a logarithmic scale. Source data are available online for this figure.

© The Author(s)

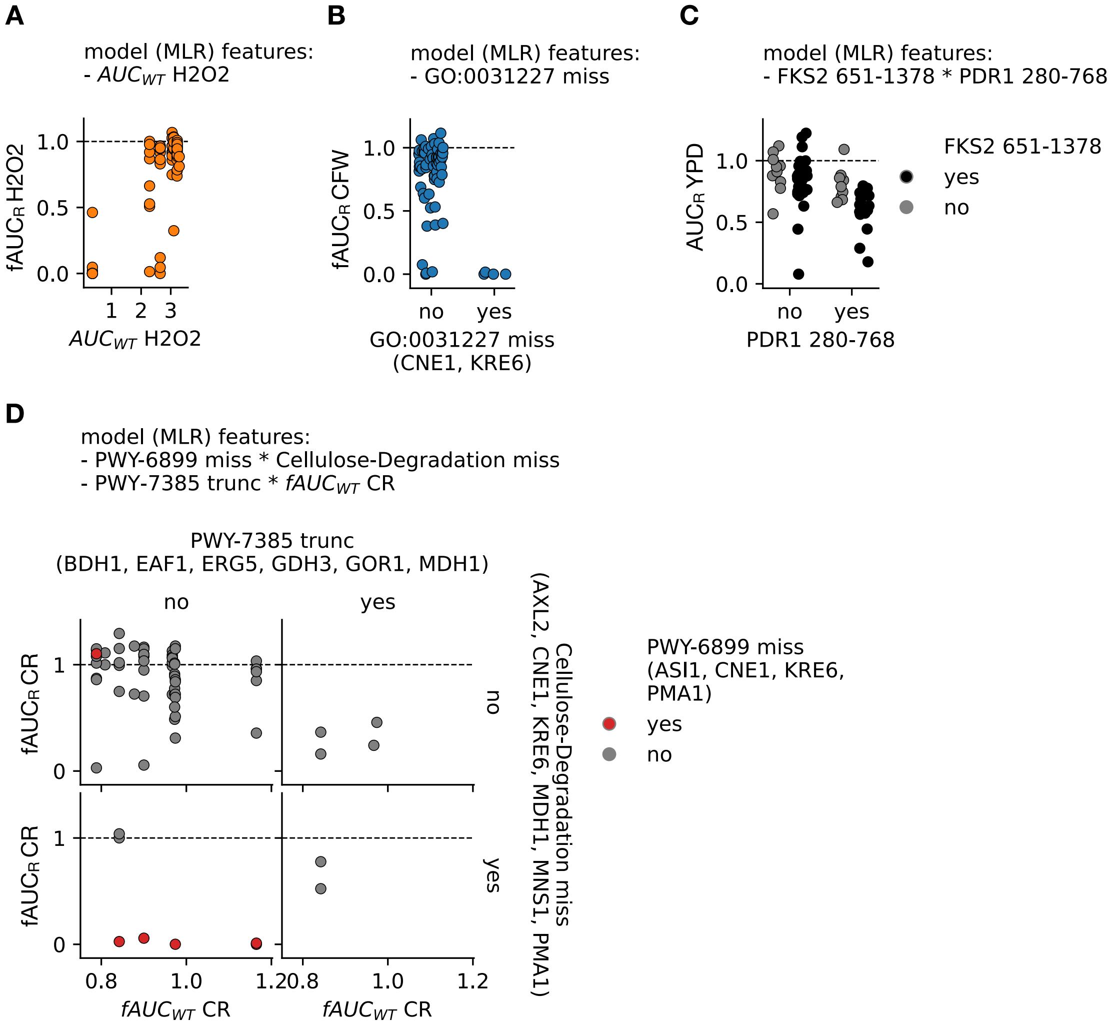

Figure EV5. Top model results for phenotypes with lower predictive accuracy.

Panels **A–D** are scatterplots equivalent to those in Fig. 5E–G, but for H_2O_2, CFW, YPD, and CR, respectively See Fig. 5D for reference about the pathway-related figures. Source data are available online for this figure.

© The Author(s)

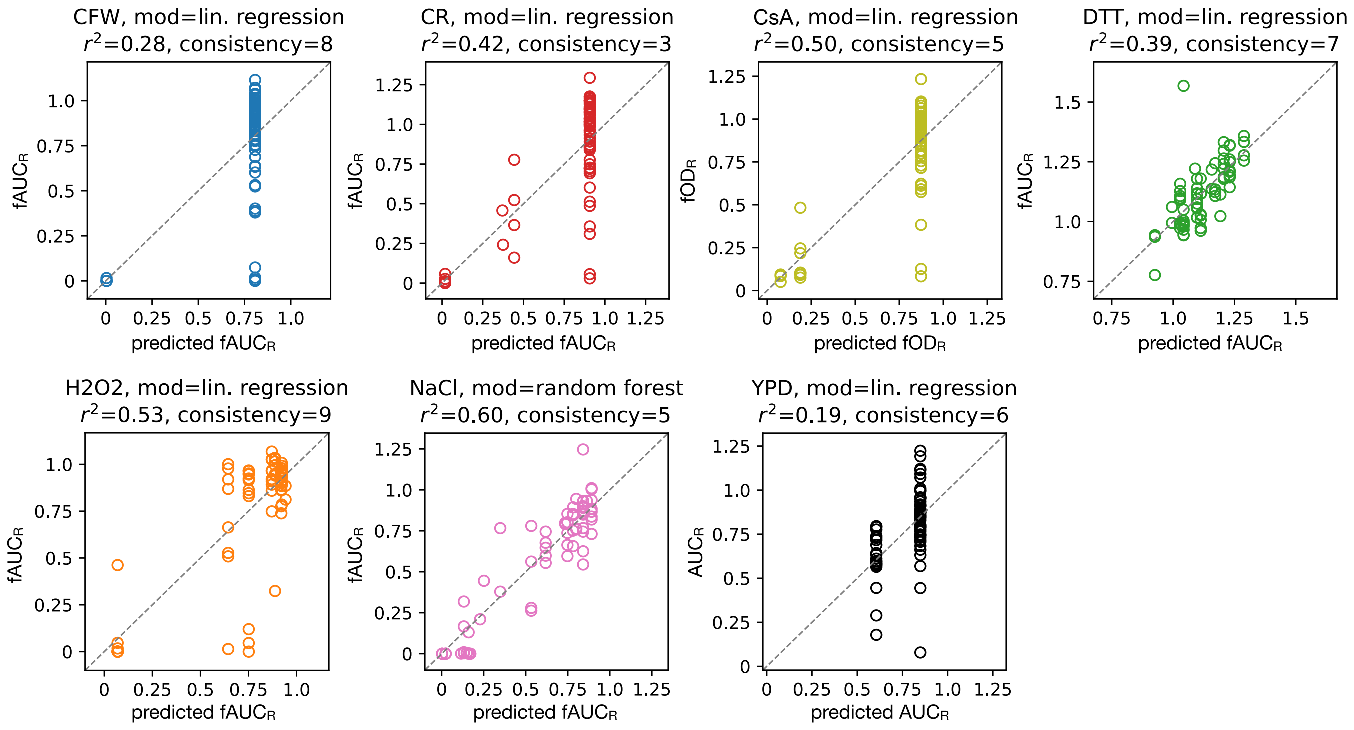

Figure EV6. Predictive performance of the top models.

Across all different strains, in each condition, the predicted trade-off intensity (x-axis) vs the actual one (y-axis) for the top models (see Fig. 5C). The title indicates the condition, type of model used (linear regression or random forest), the model performance (r^2) and the model consistency. Source data are available online for this figure.
